# Supplementary material for: Bioactive Sesquiterpenoids from Santolina chamaecyparissus L. Flowers: Chemical Profiling and Antifungal Activity Against Neocosmospora Species
Source: Plants (Basel). 2025 Jan 16;14(2):235. doi: 10.3390/plants14020235 (PMC11768361; doi:10.3390/plants14020235)
Supplement: Supplementary file 1 [file plants-14-00235-s001.zip › plants-3416134-supplementary.pdf]

Bioactive Sesquiterpenoids from *Santolina chamaecyparissus* L. Flowers: Chemical Profiling and Antifungal Activity Against *Neocosmospora* Species

E. Sánchez-Hernández, J. Martín-Gil, V. González-García,
J. Casanova-Gascón, P. Martín-Ramos

SUPPORTING INFORMATION

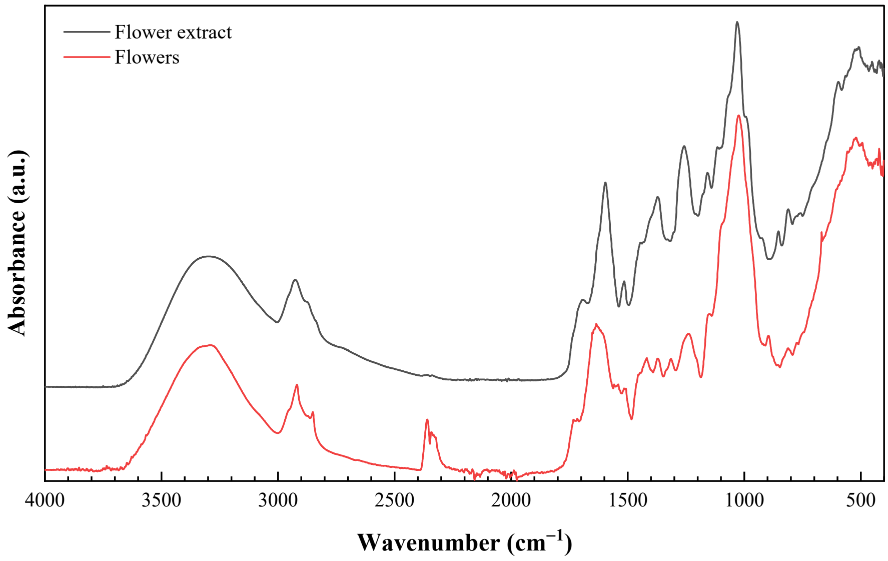

Figure S1. FTIR spectra of *Santolina chamaecyparissus* L. flowers and flower hydromethanolic extract.

Table S1. Main chemical compounds identified by GC-MS in *S. chamaecyparissus* flower extract.

RT (min)	Area (%)	Assignment	Qual
3.1225	0.0875	Formic acid	50
3.2531	3.7233	Acetic acid	91
3.4786	1.2124	2-Propanone, 1-hydroxy-	80
3.6151	0.0684	Acetic acid, hydroxy-, methyl ester	76
5.4076	0.0709	Butyrolactone	58
5.5619	0.3921	2-Cyclopenten-1-one, 2-hydroxy-	83
6.5472	0.9665	4-Hydroxy-3-[[1,3-dihydroxy-2-propoxy]methyl]-1H-pyrazole-5-carboxamide	83
6.8083	0.2287	Eucalyptol	90
6.8974	0.0972	Cycloheptanone	80
7.0161	0.2112	Benzeneacetaldehyde	76
7.3366	0.1030	2,5-Dimethyl-4-hydroxy-3(2H)-furanone	46
7.9360	0.5190	N-(n-Propyl)acetamide	50
8.1556	0.1061	Pyrimidine-4,6-diol, 5-methyl-	74
8.9035	0.4258	Benzoic acid	97
9.0400	1.0070	endo-Borneol [= Bicyclo[2.2.1]heptan-2-ol, 1,7,7-trimethyl-, (1S-endo)-]	90
9.3546	0.1867	(+)-4-Carene	91
9.5089	1.6167	Catechol	97
9.6929	0.6706	1,4:3,6-Dianhydro-α-d-glucopyranose	60
10.7197	0.9880	Hydroquinone	94
11.0046	0.3423	2-Methoxy-4-vinylphenol	93
11.5625	0.2326	Eugenol	98
11.6219	0.2295	n-Decanoic acid	74
12.0136	1.1173	4-Ethylcatechol	87
12.1264	0.1156	2-Cyclopenten-1-one, 2,3,4,5-tetramethyl-	46
12.1857	0.1045	4-Hydroxy-2-methoxybenaldehyde	93
12.2688	0.0871	7-norbornenol [= Bicyclo[2.2.1]hept-2-en-7-ol]	38
12.8327	11.9994	2,1,3-Benzothiadiazole	38
13.6577	0.6159	D-Allose	59
13.6933	0.3523	3,4-Altrosan	52
13.7467	0.2333	L-Lyxose	47
14.1147	1.3782	Cyclohexanemethanol, 4-ethenyl-α,α,4-trimethyl-3-(1-methylethenyl)-, [1R-(1α,3α,4β)]-	93
14.5480	0.0745	Cyclohexane, 1,5-diethenyl-2,3-dimethyl-, (1α,2α,3α,5α)-	50
14.6014	0.0726	7-Oxabicyclo[4.1.0]heptane, 1-methyl-4-(2-methyloxiranyl)-	47
14.7438	2.2249	Ledol [= 1H-Cycloprop[e]azulen-4-ol, decahydro-1,1,4,7-tetramethyl-, [1aR-(1aα,4β,4aβ,7α,7aβ,7bα)]-]	98
14.9100	0.4877	Sucrose	38
15.0822	27.4348	Cedren-13-ol, 8-	89
15.2424	1.4099	Isoledene [= 1,1,4,7-tetramethyl-1a,2,3,4,5,6,7,7b-octahydrocyclopropa[e]azulene]	64
15.4383	1.4855	1H-Indene, 1-ethylideneoctahydro-7a-methyl-, (1Z,3aα,7aβ)-	93
15.6757	0.5014	Longipinocarvone	56
15.7528	1.5125	Alloaromadendrene oxide-(2)	83
15.7944	0.6213	Naphthalene, 1,2,3,4,4a,5,6,7-octahydro-4a-methyl-	50
16.0734	2.0054	Aziridine, 2-methyl-2-(2,2,4-trimethyl-4-phenylpentyl)-	43
16.3048	0.7562	Spiro[2.5]octane, 5,5-dimethyl-4-(3-oxobutyl)-	35
16.5601	0.3364	Alloaromadendrene [= (1aR,4aS,7R,7aR,7bS)-1,1,7-trimethyl-4-methylidene-2,3,4a,5,6,7,7a,7b-octahydro-1aH-cyclopropa[e]azulene]	53
16.6135	0.6934	Isoquinolin-6,7-diol, 1-methyl-	35
16.6847	1.1801	3-buten-2-one, 4-(5,5-dimethyl-1-oxaspiro[2.5]oct-4-yl)	38
16.7381	0.3638	Retinal	32
16.8984	1.0094	Perhydrocyclopropa[e]azulene-4,5,6-triol, 1,1,4,6-tetramethyl	35
17.1536	1.1306	12-Oxatetracyclo[4.3.1.1(2,5).1(4,10)]dodecane, 11-isopropylidene-	42
17.2426	1.5373	2-Naphthalenemethanol, decahydro-α,α,4a-trimethyl-8-methylene-, [2R-(2α,4aα,8aβ)]-	58
17.3020	3.0574	(-)-Spathulenol [= (1aR,4aR,7S,7aR,7bR)-1,1,7-trimethyl-4-methylidene-1a,2,3,4a,5,6,7a,7b-octahydrocyclopropa[h]azulen-7-ol]	99

RT (min)	Area (%)	Assignment	Qual
17.6996	0.3661	Longifolene [= 4,8,8-trimethyl-9-methylidenedecahydro-1,4-methanoazulene]	60
17.7352	0.3316	9-Isopropyl-1-methyl-2-methylene-5-oxatricyclo[5.4.0.0(3,8)]undecane	80
18.0379	1.0511	2-Cyclopenten-1-one, 3-methyl-2-(2,4-pentadienyl)-, (Z)-	25
18.1092	0.7733	cis-α-Copaene-8-ol	50
18.5068	0.6494	Aromadendrene [= 1aR-1aα,4aα,7α,7aβ,7bα-Decahydro-1,1,7-trimethyl-4-methylene-1H-cycloprop[e]azulene]	49
18.8392	1.3693	2-Oxabicyclo[2.2.2]octan-6-ol, 1,3,3-trimethyl-, acetate	38
18.9460	1.4521	Epiglobulol [= (1aR,4S,4aR,7R,7aS,7bS)-1,1,4,7-tetramethyl-2,3,4a,5,6,7,7a,7b-octahydro-1aH-cyclopropa[e]azulen-4-ol]	50
19.1003	1.3108	1H-Benzocyclohepten-7-ol, 2,3,4,4a,5,6,7,8-octahydro-1,1,4a,7-tetramethyl-, cis-	27
19.5693	6.3413	Ledene [= 1H-Cycloprop[e]azulene, 1a,2,3,5,6,7,7a,7b-octahydro-1,1,4,7-tetramethyl-, [1aR-(1aα,7α,7aβ,7bα)]-] oxide-(II)	92
19.8363	0.4439	5,6-Azulenedimethanol, 1,2,3,3a,8,8a-hexahydro-2,2,8-trimethyl-, (3aα,8β,8aα)-	35
19.8838	0.6876	Longiborneol [= 1,4-Methanoazulen-7-ol, decahydro-4,8,8,9-tetramethyl-, (+)-]	56
20.1628	0.8508	1,3,5,7-Tetramethyl-adamantane	50
20.2162	0.8754	Alloaromadendrene oxide-(1)	80
20.2874	0.4805	7-(1,3-Dimethylbuta-1,3-dienyl)-1,6,6-trimethyl-3,8-dioxatricyclo[5.1.0.0(2,4)]octane	80
20.3943	1.8130	Spiro[2,4,5,6,7,7a-hexahydro-2-oxo-4,4,7a-trimethylbenzofuran]-7,2'-(oxirane)	64
20.4655	0.5449	Indole-2-one, 2,3-dihydro-N-hydroxy-4-methoxy-3,3-dimethyl-	41
20.6673	0.3016	5H-Benzo[b]pyran-8-ol, 2,3,5,5,8a-pentamethyl-6,7,8,8a-tetrahydro-	12
20.7682	2.2728	Retinol, acetate	52
21.5813	0.7008	3-Buten-2-one, 4-(2,6,6-trimethyl-1-cyclohexen-1-yl)-	45

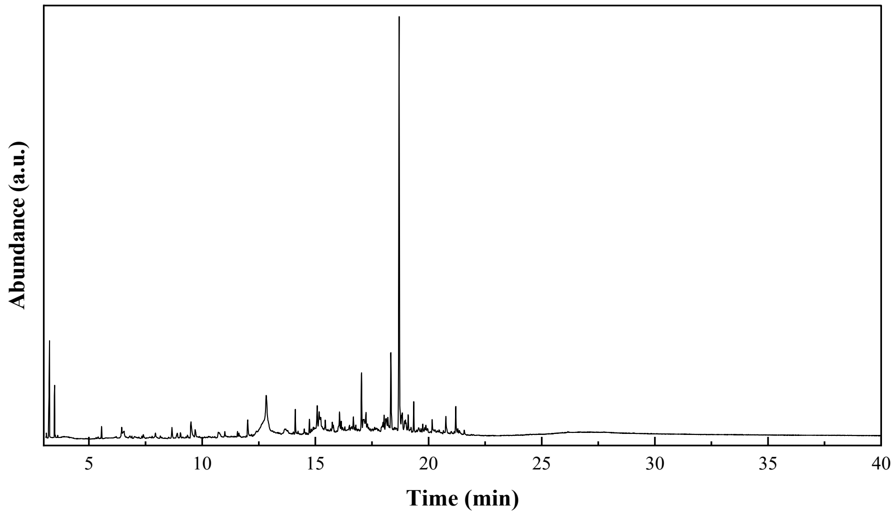

Figure S2. GC–MS chromatogram of *S. chamaecyparissus* flower hydromethanolic extract.

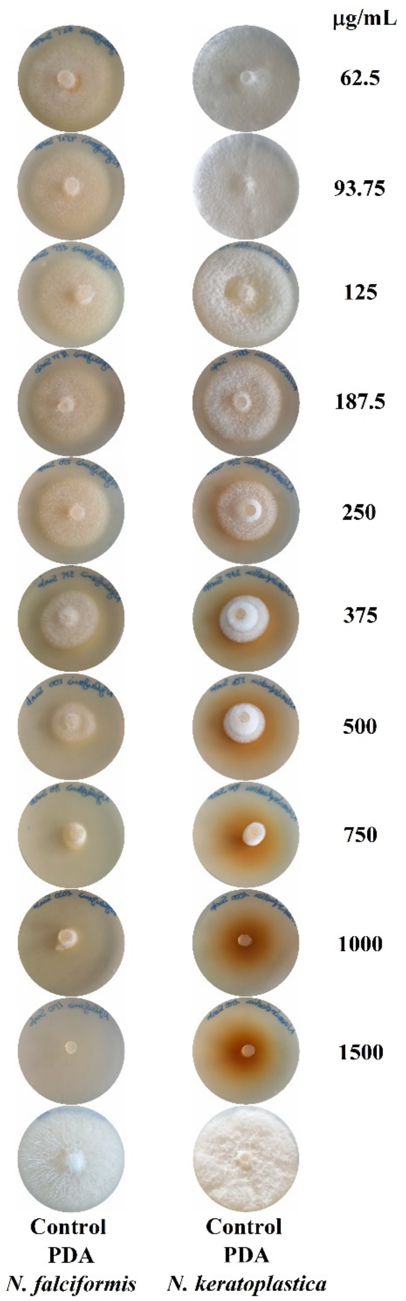

Figure S3. *In vitro* antifungal activity assays showing growth inhibition of *Neocosmospora falciformis* (left) and *N. keratoplastica* (right) by *S. chamaecyparissus* extract at different concentrations (15.62–1500 µg·mL^{-1}). Representative plates from one replicate per treatment are shown. Control plates (PDA without extract) are not shown (mycelial growth reached 75 mm diameter).

Table S2. Antimicrobial activity of *S. chamaecyparissus* extracts and essential oils previously reported in the literature.

Collection site	Part of plant	Extraction procedure	Microorganisms	Efficacy	Ref.
Spain	Flowers	Methanol:water (1:1, *v/v*)	*N. falciformis*	MIC = 1500 μg·mL^{-1}	This work
			N. keratoplastica	MIC = 1000 μg·mL^{-1}	
Tunisia	Flowers or roots	Essential oil by hydrodistillation	*Escherichia coli*	MIC = 1.25 μg·mL^{-1}	[1]
			Pseudomonas aeruginosa	MIC = 0.625–5 μg·mL^{-1}	
			Proteus mirabilis	MIC ≥ 10 μg·mL^{-1}	
			Citrobacter freundii	MIC = 5–10 μg·mL^{-1}	
			Enterococcus faecalis	MIC = 0.625–2.5 μg·mL^{-1}	
			Staphylococcus aureus	MIC = 1.25–2.5 μg·mL^{-1}	
			Trichophyton rubrum	MIC = 500–750 μg·mL^{-1}	
			Microsporum canis	MIC = 750–1000 μg·mL^{-1}	
			Epidermophyton floccosum	MIC = 500–1000 μg·mL^{-1}	
			Candida albicans	MIC = 1000 μg·mL^{-1}	
			Scytalidium dimidiatum	MIC = 500–1000 μg·mL^{-1}	
			Scopulariopsis brevicaulis	MIC = 500–1000 μg·mL^{-1}	
			Aspergillus fumigatus	MIC = 500–1000 μg·mL^{-1}	
Turkey	Flowers	Essential oil by microwave-assisted distillation system	*Escherichia coli*	MIC = 2000 μg·mL^{-1}	[2]
			Streptococcus mutans	MIC = 500 μg·mL^{-1}	
			Bacillus cereus	MIC = 500 μg·mL^{-1}	
			Bacillus subtilis	MIC = 1000 μg·mL^{-1}	
			Candida albicans	MIC = 1000 μg·mL^{-1}	
India	Flowers	Essential oil by steam distillation	*Candida albicans*	MIC = 62.5 μg·mL^{-1}	[3]
Algeria	Flowers	Essential oil by hydro-distillation at 20%	*Pseudomonas aeruginosa*	IZ = 21.1 mm	[4]
			Escherichia coli	IZ = 18.3 mm	
			Klebsiella pneumoniae	IZ = 34.1 mm	
			Bordetella bronchiseptica	IZ = 17.2 mm	
			Staphylococcus aureus	IZ = 24.1 mm	
			Staphylococcus epidermis	IZ = 19.0 mm	
			Enterococcus faecalis	IZ = 20.3 mm	
			Micrococcus luteus	IZ = 16.1 mm	
			Candida albicans	IZ = 35.0 mm	
			Saccharomyces cerevisiae	IZ = 20.6 mm	

Table S2 (cont.). Efficacy of plant extracts and essential oils reported in the literature against the phytopathogens under study.

Collection site	Part of plant	Extraction procedure	Microorganisms	Efficacy	Ref.
Algeria	Flowers	Essential oil by hydro-distillation or ethanol 95% extraction	*Staphylococcus aureus*	IZ = 11–37 mm	[5]
			Bacillus subtilis	IZ = 11–36 mm	
			Escherichia coli	IZ = 9 mm	
			Candida albicans	IZ = 9–17 mm	
			Saccharomyces cerevisiae	IZ = 11–15 mm	
Syria	Leaves	Essential oil by hydro-distillation	*Bacillus subtilis*	MIC = 800 μg·mL^{-1}	[6]
			Staphylococcus aureus	MIC = 1600 μg·mL^{-1}	
			Streptococcus pyogenes	n.a.	
			Micrococcus luteus	MIC = 1600 μg·mL^{-1}	
			Escherichia coli	MIC = 1600 μg·mL^{-1}	
			Salmonella typhimurium	n.a.	
			Pseudomonas aeruginosa	MIC = 800 μg·mL^{-1}	
			Klebsiella pneumoniae	MIC = 400 μg·mL^{-1}	
			Proteus vulgaris	n.a.	
			Vibrio parahaemolyticus	n.a.	
			Fusarium solani	MIC = 800 μg·mL^{-1}	
			Aspergillus flavus	n.a.	
			Penicillium variabile	n.a.	
			Mucor sp.	MIC = 1600 μg·mL^{-1}	
			Rhizopus sp.	MIC = 3200 μg·mL^{-1}	
			Candida albicans	MIC = 400 μg·mL^{-1}	
Morocco	Leaves	aqueous extraction	*Fusarium oxysporum* f. sp. *lentis*	MIC > 20,000 μg·mL^{-1}	[7]
		Commercial essential oil at 30%	*Botrytis cinerea*	n.a.	[8]
			Sclerotinia sclerotiorum	PMIG = 54.50%	
			Fusarium oxysporum	PMIG = 67.65%	
			Phytophthora parasitica	PMIG = 72.02%	
			Alternaria brassicae	PMIG = 10.65%	
			Cladobotryum mycophilum	PMIG = 68.69%	
			Pythium aphanidermatum	n.a.	

IZ: inhibition zone; MIC: minimum inhibitory concentration; n.a.: no activity at the highest concentration tested; PMGI: percentage of mycelial growth inhibition.

Table S3. Efficacy of plant extracts and essential oils reported in the literature against the *Neocosmospora* species under study.

Pathogen	Source/Extraction Medium	Plant	Efficacy	Ref.
N. falciformis	Methanol/water (1:1 *v/v*)	*S. chamaecyparissus* flowers	MIC = 1500 µg·mL^{-1}	This work
	Methanol/water (1:1 *v/v*)	*Armeria maritima* flowers	MIC = 1000 µg·mL^{-1}	[9]
	Essential oil	*Citronella* spp.	PMIG = 100% at 2%	[10]
		Melaleuca spp.	PMIG = 40.1% at 2.5%	
	Essential oil	*Cinnamomum aromaticum*	PMIG = 100% at 0.0625%	[11]
		Cymbopogon flexuosus	PMIG = 100% at 0.125%	
		Ocimum basilicum	PMIG = 64.2–72% at 0.5%	
	Ethanol 95%/water solution (1:1 *v/v*)	*Hibiscus sabdariffa*	MIC = 10,000 µg·mL^{-1}	[12]
		Syzygium aromaticum	MIC = 1000 µg·mL^{-1}	
		Curcuma longa	MIC = 10,000 µg·mL^{-1}	
		Cymbopogon citratus	MIC > 10,000 µg·mL^{-1}	
N. keratoplastica	Methanol/water (1:1 *v/v*)	*S. chamaecyparissus* flowers	MIC = 1000 µg·mL^{-1}	This work
	Methanol/water solution (1:1 *v/v*)	*Armeria maritima* flowers	MIC = 1500 µg·mL^{-1}	[9]
	Essential oil	*Trachyspermum ammi* seeds	n.a.	[13]
	Essential oil	*Kaempferia parviflora* rhizome	IZ = 17–18 mm	[14]
	Essential oil	*Pogostemon cablin* flowers + leaves	n.a. at 500 µg·mL^{-1}	[15]
	Essential oil	*Origanum vulgare* subsp. *hirtum*	MIC = 800 µg·mL^{-1}	[16]
	Essential oil	*Matricaria chamomilla* flowers	MIC = 20 µg·mL^{-1}	[17]

IZ: inhibition zone; MIC: minimum inhibitory concentration; n.a.: no activity at the highest concentration tested; PMGI: percentage of mycelial growth inhibition.

References (reference numbers do not match those that appear in the main document):

1. Bel Hadj Salah-Fatnassi, K.; Hassayoun, F.; Cheraif, I.; Khan, S.; Jannet, H.B.; Hammami, M.; Aouni, M.; Harzallah-Skhiri, F. Chemical composition, antibacterial and antifungal activities of flowerhead and root essential oils of *Santolina chamaecyparissus* L., growing wild in Tunisia. *Saudi J. Biol. Sci.* **2017**, *24*, 875-882, doi:10.1016/j.sjbs.2016.03.005.
2. Süfer, Ö.; Ceylan, A.; OnbaŞili, D.; Çelik Yuvali, G.; Bozok, F. Chemical compounds and biological activity of Turkish *Santolina chamaecyparissus* L. Essential oil by microwave assisted distillation. *Kastamonu Univ. Orman Fak. Derg.* **2021**, *21*, 165-175, doi:10.17475/kastorman.1000463.
3. Suresh, B.; Sriram, S.; Dhanaraj, S.A.; Elango, K.; Chinnaswamy, K. Anticandidal activity of *Santolina chamaecyparissus* volatile oil. *J. Ethnopharmacol.* **1997**, *55*, 151-159, doi:10.1016/s0378-8741(96)01490-0.
4. Djeddi, S.; Djebile, K.; Hadjbourega, G.; Achour, Z.; Argyropoulou, C.; Skaltsa, H. In vitro antimicrobial properties and chemical composition of *Santolina chamaecyparissus* essential oil from Algeria. *Nat. Prod. Commun.* **2012**, *7*, 937-940, doi:10.1177/1934578X1200700735.
5. Chirane, M.S.; Benchabane, O.; Bousbia, N.; Zenia, S. Antioxydant and antimicrobial activities of essential oil and ethanol extract of *Santolina chamaecyparissus* L. *Rev. Agrobiol* **2019**, *9*, 1660-1668.
6. Khubeiz, M.J.; Mansour, G. In vitro antifungal, antimicrobial properties and chemical composition of *Santolina chamaecyparissus* essential oil in Syria. *Int. J. Toxicol. Pharm. Res* **2016**, *8*, 11.
7. Aourach, M.; Barbero, G.F.; González de Peredo, A.V.; Diakite, A.; El Boukari, M.; Essalmani, H. Composition and antifungal effects of aqueous extracts of *Cymbopogon citratus, Laurus nobilis* and *Santolina chamaecyparissus* on the growth of *Fusarium oxysporum* f. sp. *lentis*. *Arch. Phytopathol. Pflanzenschutz* **2021**, *54*, 2141-2159, doi:10.1080/03235408.2021.1922169.
8. Diánez, F.; Santos, M.; Parra, C.; Navarro, M.J.; Blanco, R.; Gea, F.J. Screening of antifungal activity of 12 essential oils against eight pathogenic fungi of vegetables and mushroom. *Lett. Appl. Microbiol.* **2018**, *67*, 400-410, doi:10.1111/lam.13053.
9. Sánchez-Hernández, E.; Martín-Ramos, P.; Navas Gracia, L.M.; Martín-Gil, J.; Garcés-Claver, A.; Flores-León, A.; González-García, V. *Armeria maritima* (Mill.) Willd. flower hydromethanolic extract for Cucurbitaceae fungal diseases control. *Molecules* **2023**, *28*, 3730, doi:10.3390/molecules28093730.
10. Medeiros Araújo, M.B. Species of Fusarium causing peduncular rot in melon in Brazil and alternative management methods. Universidade Federal Rural do Semi-Árido, Mossoró, Brazil.
11. de Medeiros, A.S.; Costa Alves, T.R.; dos Santos Silva, J.L.; de Moura, A.P.; de Lima, J.S.S.; de Souza, J.J.F.; de França, M.A.V.; da Costa Fernandes, J.; Bezerra Evangelista, L.F.; de Queiroz Ambrósio, M.M. Thermotherapy combined with alternative products in the management of melon rot caused by Fusarium falciforme. *Eur. J. Plant Pathol.* **2023**, 10.1007/s10658-023-02805-w, doi:10.1007/s10658-023-02805-w.
12. Srihom, C.; Boonyuen, N.; Khewkhom, N.; Leesutthiphonchai, W.; Nuankaew, S.; Suetrong, S.; Chuaseeharonnachai, C.; Piasai, O. Potential of herb crude extracts against Thai isolates of *Fusarium* wilt pathogens. *Curr. Res. Environ. Appl. Mycol.* **2021**, *11*, 570-584.
13. Dutta, P.; Sarma, N.; Saikia, S.; Gogoi, R.; Begum, T.; Lal, M. Pharmacological activity of *Trachyspermum ammi* L. seeds essential oil grown from Northeast India. *J. Essent. Oil-Bear. Plants* **2022**, *24*, 1373-1388, doi:10.1080/0972060x.2022.2028681.
14. Begum, T.; Gogoi, R.; Sarma, N.; Pandey, S.K.; Lal, M. Direct sunlight and partial shading alter the quality, quantity, biochemical activities of *Kaempferia parviflora* Wall., ex Baker rhizome essential oil: A high industrially important species. *Ind Crops Prod* **2022**, *180*, 114765, doi:10.1016/j.indcrop.2022.114765.
15. Pandey, S.K.; Gogoi, R.; Bhandari, S.; Sarma, N.; Begum, T.; Munda, S.; Lal, M. A comparative study on chemical composition, pharmacological potential and toxicity of *Pogostemon cablin* Linn., (Patchouli) flower and leaf essential oil. *J. Essent. Oil-Bear. Plants* **2022**, *25*, 160-179, doi:10.1080/0972060x.2021.2013325.
16. Krumova, E.; Nikolova, M.; Miteva-Staleva, J.; Kostadinova, N.; Abrashev, R.; Dishliyska, V.; Berkov, S.; Mutafova, B.; Angelova, M. Bio-efficacy of the essential oil isolated from *Origanum vulgare* subsp. *Hirtum* against fungal pathogens of potato. *C. R. Acad. Bulg. Sci.* **2021**, *74*, 1571, doi:10.7546/CRABS.2021.10.18.
17. Sarma, N.; Gogoi, R.; Begum, T.; Lal, M.; Perveen, K.; Alsahikh, N.A.; Alsulami, J.A. A study on the chemical profile of cultivated chamomile (*Matricaria chamomilla* L.) flower essential oil from North East India with special emphasis on its pharmacological importance. *J. Essent. Oil-Bear. Plants* **2023**, *26*, 745-760, doi:10.1080/0972060x.2023.2239289.
